# Influence of a double-stage solar tunnel dryer on the preservation of quality characteristics and the Modelling of colour variations in red chili peppers

**DOI:** 10.1016/j.heliyon.2024.e36857

**Published:** 2024-08-24

**Authors:** Eshetu Getahun, Mulugeta A.Delele, Nigus Gabbiye, Solomon Workneh, Maarten Vanierschot

**Affiliations:** a Bahir Dar Energy Center, Bahir Dar Technology Institute, Bahir Dar University, Ethiopia; b KU Leuven, Department of Mechanical Engineering, Leuven, Belgium; c Material Science, Innovation and Modelling (MaSIM), North-West University, Mmabatho, South Africa; d Faculty of Chemical and Food Engineering, Bahir Dar Technology Institute, Bahir Dar University, Ethiopia

**Keywords:** Chili pepper quality, Colour change model, Solar tunnel drying, Double stage dryer

## Abstract

In this work, the influence of two-stage solar tunnel drying on the preservation of quality attributes of both chili pepper varieties, *Mareko Fana* (MF) and *Bako Local* (BL), was studied. Both varieties were dried in different drying modalities, i.e. solar tunnel and open sun drying. The quality attributes were evaluated during the drying process using FTIR and spectrophotometry techniques. Different colour evolution models were implemented and a suitable model was selected. The results showed that after drying of the *Mareko Fana* chili variety, the lightness retention was found in the range of 81 %–89 %, the redness retention between 25 % and 42 % and the yellowness retention between 14 % and 38 %. For the *Bako Local* variety, the values were in the range of 78 %–82 %, 42 %–60 % and 36 %–55 %, respectively. The first order model gave a higher correlation coefficient (R^2^ > 0.96), which indicated the suitability of the model for predicting the color variation for both varieties. The volatile aromatic compounds in the chili peppers were greatly lost during open sun drying, while solar tunnel drying maintained these compounds for both varieties. However, during the solar tunnel drying process, a significant amount of alkyl halides or alkyl-chlorides were lost. The amount of dihydrocapsaicin and capsaicin from the chili peppers ranged from 10,172 μg/kg to 16,313 μg/kg and 16,676 μg/kg to 27,189 μg/kg for both dihydrocapsaicin and capsaicin, respectively. In tunnel, open-air, and uncontrolled open-air solar drying, the MF chili variety lost copsaicinoid content by 3.4 %, 14.8 %, and 38.3 %, respectively; in the BL variety, comparable losses were 1.8 %, 4.4 %, and 13.6 %. A minimum loss of ascorbic acids was recorded during solar tunnel drying. The results showed that well-designed double stage solar tunnel dryers are important for effective drying processes that preserve quality attributes of the chili pepper.

**Table:** Nomenclature

$C_{o}$	Initial value of surface colour parameters (L*, a* & b*)
$C_{t}$	Surface colour parameters value at time t
$C_{f}$	Surface colour value of the final dried sample
K	Rate constant (h ^1^)
t	Drying time (s)
α	Weibull scale parameter (h)
β	Shape dimensionless parameter
A	Absorbance (dimensionless)
b	Path length through the sample (1 cm)
ε	Molar absorbance coefficient (liter mol^−1^)
c	Molar concentration (mol liter^−1^)
S	Slope of the calibration curve
σ	Standard deviation of y-intercepts
$R^{2}$	Coefficient of determination
${MR}_{\exp}$	Experimental moisture ratio
${MR}_{pre}$	Predicted moisture ratio
${\bar{Mt}}_{pre,i}$	The average value of predicted moisture ratio
N	Number of observations
z	Number of drying constants
$L^{*}$	Whiteness, L*o fresh whiteness
$a^{*}$	Redness, a*o, fresh redness
$b^{*}$	Blueness, b*o fresh blueness
x	Uncertainty properties
y	Characteristic equation
T	Transmittance (%)
**Abbreviations**	
MF	Mareko Fana chili variety
BL	Bako Local chili variety
MR	Mean moisture ratio
RMSE	Root mean square error
MBE	Mean biased error
RH	Relative humidity of ambient air (%)
LOD	Limit of detection
LOQ	Limit of quantification
NDC	Nordihydrocapsaicin
CAP	Capsaicin
DC	Dihydrocapsaicin
HCAP1-2,	Homocapsaicin
HDC1-2,	Homodihydrocapsaicin
SHU	Scoville heat unit
**Greek Letters**	
$\chi^{2}$	Reduced chi-square
**Subscripts**	
pre	Predicted
exp	Experimental
t	Time t
o	Initial
f	Final

## 1 Introduction

Nowadays, climate change and the increasing demand for food worldwide have resulted in more advanced research on the preservation of various food items based on low cost and affordable preservation mechanisms using renewable energy sources. Otherwise, several fruits and vegetables could be lost as a result of glut production. Amongst other different food products, chili pepper (*Capsicum annuum*), originally from tropical regions, is susceptible to high postharvest losses due to improper drying. Due to its colour, pungency and culinary value, chili pepper is used widely as a condiment and it is the second most consumed vegetable worldwide next to tomato [1]. Chili peppers are characterized by high levels of bioactive compounds, antioxidants and phytochemicals, such as carotenoids, β-carotene (pro-vitamin-A), ascorbic acid (vitamin C), phenolic compounds and minerals. Hence, they can serve as healthy food or have a good pharmacological potential [2,3]. Moreover, they also contain thousands of other metabolites, including fatty oils, water, resin, carotenoids, fibres, protein, minerals, and volatile compounds, which influence the peppers’ nutritional value, colour, taste and aroma during solar drying and storage [4,5]. Carotenoids are an important source of colour during the ripening stage [6]. The important source of pungency of chili pepper is due to capacious (composed of capsaicin, dihydrocapsacinoids, nordihydrocapsacinoids, homocapsaicin and homohydrocapsaicin) and they are responsible for the hotness of the chili peppers [7]. Capsaicin, dihydrocapsacinoids and nordihydrocapsacinoids in the group of capsacinoids represent 69 %, 22 % and 7 %, respectively and others represent only 1 % [8]. Thus, the capsaicin and dihydrocapsacinoids dominantly represent capsacinoids (about 90 %). Pungency is measured by a Scoville Organoleptic Scale that ranges from 0 to 500,000 Scoville heat units (SHU) and it is classified as non-pungent (0–700 SHU), mildly (700–3000 SHU), moderately (3000–25,000 SHU), highly (25, 000–70,000 SHU), and very highly pungent (>80,000 SHU) [9,10]. Despite these rich bioactive compounds, chili peppers are very perishable commodities since their moisture content is higher than 80 %. This significantly limits their shelf-life and makes them more susceptible to micro-organisms during storage and transport [11]. Moreover, chili pepper production is highly seasonal and only available in large amounts during the harvesting periods of the year, especially in developing countries. Many developing countries have experienced a significant increase in post-harvest losses of agricultural commodities. Numerous studies shown that postharvest losses of fruits and vegetables in developing countries range from 30 to 40 %, with Ethiopia seeing losses of about 50 % [12,13].

Therefore, appropriate drying technologies are very essential during the high production season to enable the availability of peppers throughout the entire year.

Among other food preservation techniques, drying is one of the most common methods and can reduce overall transportation cost in addition to an increase in shelf-life by reducing the activities of enzymes and microorganisms, both causing spoilage of the food items [14]. There are several different types of drying technologies for fruits and vegetables such as open sun drying [15], solar drying [12,16], tray drying [17], vacuum drying [18] or fluidized bed drying [19]. Open sun and solar drying are very common in developing countries since their energy source is low cost, sustainable and renewable. However, open sun drying requires long drying times, generally between five to seven days, depending on the region of drying [20]. In spite of its economic advantage, open sun drying has several other limitations such as an uncontrollable drying temperature, a large drying space, much hand labour involved and an exposure of products to contamination by fungi, birds and insects, which results in colour and bioactive compound degradation of the final product [21]. To alleviate the limitations of open sun drying, different solar dryers have been designed and optimized to reduce the drying time, drying area, and labour force and to prevent products from contamination, all resulting in an improved product quality.

One of the advantages of solar drying is that it is highly effective and efficient to reduce postharvest losses, to increase product quality and to balance the shortages in supply during non-harvesting seasons. It is also a more convenient alternative energy usage for rural societies who are living far away from the electric grid. During solar drying, the product is heated either through the passage of hot air along the product (indirect drying), by directly absorbing solar energy (direct drying) or a combination of both [22]. The effects of these drying methods on the bioactive compounds of chili peppers are reported by Montoya-Ballesteros et al. [3]. Compared to open air/sun drying, indirect solar drying provides more control over product quality and prevents contamination and deterioration of bioactive compounds. However, at long drying times or high drying temperatures, the solar drying process may cause some losses of colour and bioactive compounds. These main factors can degrade bioactive compounds and nutrients, such as vitamin E, ascorbic acid, phenol compounds, carotenoids, and flavonoids [2,23]. Therefore, in solar drying, it is necessary to obtain a steady operating temperature as soon as possible, since drying at high temperature and long drying time deteriorates the products’ quality, more specifically bioactive compounds such as colour, flavour, pungency and other antioxidants [24]. In the work of Getahun et al. [12], an indirect double stage solar tunnel drier was developed to investigate the drying properties of two chili pepper cultivars. It was looked at how the density of the chili layer affected the drying period and moisture loss. Also, analysis was done on carbon footprint and the drying efficiency was studied. To fit the experimental results, thirteen thin layer-drying models have been examined. The collectors' efficiencies varied from 66.44 % to 76.53 %. The dryer has a specific energy consumption range of 2.1–2.44 kWh/kg and an estimated CO2 emission of 53.8 kg/kWh. However, detailed quality attributes of the dried chili peppers were not characterized and the best colour evolution model was not determined. Thus, the double stage solar tunnel dryer was also used in this study to assess and model the quality attributes of solar dried chili peppers and the evolution of colour variation in time.

Different drying modes, such as open sun, direct, indirect or mixed mode drying highly affect the quality of chili peppers. Reports indicated that the loss of Pro-Vitamin A and trans-β-carotene ranged from 13 % to 34 % and 16 %–34 %, respectively in direct and mixed (greenhouse) solar dryers [25]. A well-designed solar dryer has a huge effect on the preservation of quality parameters of chili pepper. Indirect solar dryers, like for instance a solar tunnel dryer, may improve the loss of Pro-Vitamin A and trans-β-carotene through passing hot air over the product in the drying chamber rather than direct absorption of solar energy. According to the investigations carried out, there is limited information on the preservation of bioactive compounds of Ethiopian chili pepper varieties during drying in an indirect type solar tunnel dryer. Therefore, the aim of this study was to investigate the effects of both drying in a double stage solar tunnel dryer and open sun drying on the retention of carotenoid, surface colour, ascorbic acid (vitamin C), and capsaicin of the *Mareko Fan* and *Bako Local* Ethiopian chili varieties.

## 2 Materials and methods

### 2.1 Materials (chili peppers and drying condition)

Fully ripe and freshly harvested chili peppers (*Mareko Fana* and *Bako Local*) were collected during the harvesting season between November to December 2019 in western Ethiopia. The peppers had an average moisture content between 79.6 and 82.4 % (w.b.), determined according to the standard method (hot air oven test) [12]. Additionally, in order to prevent moisture loss prior to solar drying, the samples were separated and stored at 6 °C until they were needed for the experiments. The drying experiment itself was conducted at Bahir Dar (Amhara Region, Ethiopia), having a humid temperature climate. The average annual temperature is 19.6^o^C (with a highest and lowest mean monthly temperature of 29.7 °C in April and 23.3 °C in July/August) and an average rainfall of 1419 mm. The daily hours of sunshine vary between 6h in July/August to 10h in January/February [26].

### 2.2 Chemicals

All the reagents, standards (capsaicin >95 %; dihydrocapsaicin about 90 % from Capsicum sp.), ascorbic acid, and other chemicals and solvents used were analytical graded or HPLC graded and purchased from Sigma-Aldrich (St. Louis, MO, USA). A 10 % acetic acid, 5 % metaphosphoric acid, 10 % thiourea, and 85 % sulphuric acid solution and 2,4-Dinitrophenylhydrazine were used to characterize the quality attributes.

### 2.3 Experimental setup

**Fig. 1:**
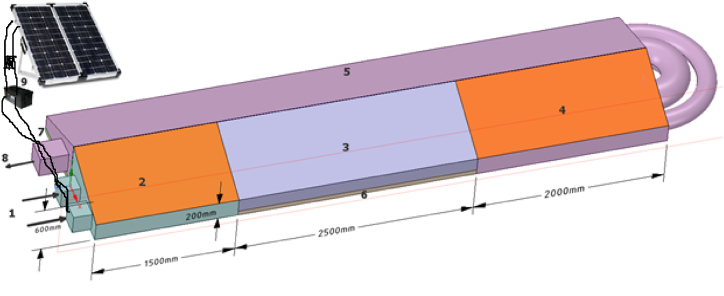
Experimental setup of indirect type solar tunnel chili drying. 1: air inlet, 2: solar collector 1, 3: dryer stage 1, 4: solar collector 2, 5: dryer stage 2, 6 &7: chili pepper bed, 8: air outlet and 9: PV module system [12].

A schematic view of the indirect type solar tunnel chili dryer is shown in Fig. 1. Drying is carried out at the same time in two different sample trays (number 6 and 7 as shown in Fig. 1) at a bed thickness of 8.5 mm. Both the samples in drying chamber 3 and 5 are chili peppers, but in between, the air is heated up again in solar collector 4. Therefore, in this study, the drying principle is a two stage, which means the drying air first goes to the first solar collector (number 2 on the figure) before going to dryer stage one (number 3). After that, the air can get heat again from the second solar collector (number 4), before finally going into to dryer stage two (number 5). The aim of this double stage dryer is to increase the overall performance of the dryer. A more detailed overview of the experimental setup and its explanation can be found in previous work [12].

### 2.4 Drying experiments

**Fig. 2:**
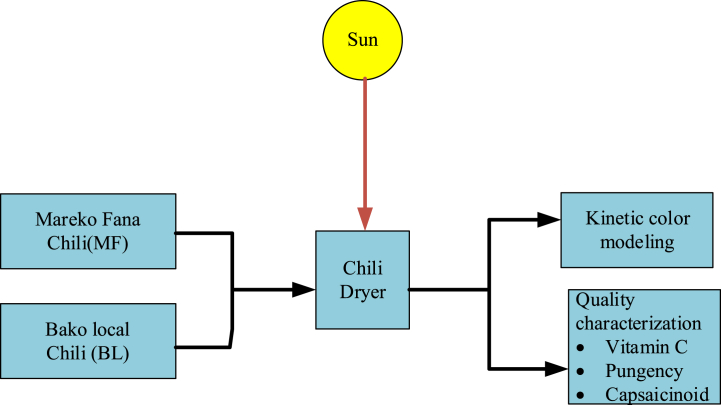
General experimental methodology during the solar tunnel chili drying process

Drying experiments were carried out between 8:00h to 16:00h every day in the period from November till December 2019. The experiments were performed at three different chili pepper bed thicknesses: a thick (8.50 kg/m^2^), a medium (7.19 kg/m^2^) and a thin layer (5.88 kg/m^2^) [12]. These thicknesses were determined after detailed optimization of the dryer using computational fluid dynamics (CFD). For all experiments, the chili peppers were loaded on the solar tunnel drying bed. The peppers were also left inside the dryer during night-time. A polyethylene plastic cover over the entire setup and a shutdown of the fan during the night prevented migration of outside moist into the dryer. Drying was continued the next day until the chili pepper moisture content reached a desirable level of 10–12 % (w.b.) [12]. Open sun drying was also carried out in parallel with solar drying to compare both drying modes with each other. In open sun drying, the chili product was dried in two ways, i.e. with and without control of the process. In the controlled manner, the chili pepper was spread out on the ground throughout the day and at nigh time the chili was stored in plastic bags to prevent moisture and other impurity contamination. However, in the uncontrolled manner, the chili pepper remained on the ground throughout the entire night. The moisture loss of the chili pepper was recorded every 2 h using a digital electronic balance (sensitivity ± 0.001 g) and samples were taken at different positions inside the dryer. A data logger (UX 100-011 Temp/RH, Onset HOBO Data Logger) was used to collect the temperature and relative humidity data of the ambient air and of the air inside the dryer at different positions along its length. A pyranometer (Apogee; Model: SP-110) with a reading accuracy of ±5 W/m^2^ measured the solar radiation intensity. The general experimental methodology is shown in detail in Fig. 2.

The effects of two different varieties of chili pepper and the thickness of the chili pepper bed on the ultimate moisture content and drying time of the peppers were examined using a full factorial design, which is an entirely randomized design. Triplicate data sets were collected for statistical analysis.

### 2.5 Characterization of quality attributes of the chili pepper

#### 2.5.1 Surface colour measurement

The colour of the chili pepper varieties was recorded at the beginning and through out the drying process by a spectrophotometer (KONICA MINOLTA, CM-600d, Japan) according to Yang et al., [27]. The CIE Lab colour parameters such as L* (whiteness or brightness), a* (redness or greenness) and b* (yellowness or blueness) were measured to describe the colour evolution of the chili pepper during the drying process. The sensor was calibrated using a standard white tile (a* = −0.08 ± 0.02, b* = 0.07 ± 0.09, L* = 97.3 ± 0.7) before measurements were commenced. Colour measurements were recorded in triplicate and mean values were taken.

#### 2.5.2 Kinetic modelling of colour evolution during drying

The kinetics of the colour degradation were determined using a zero order, first order, Weibull and fractional model [28,29] as given in Eqs. (1), (2), (3), (4):

(1) $$\text{Zero}-\text{order}\;\;\text{model}:C_{t}=C_{o}-kt\text{,}$$

(2) $$\text{First}-\;\text{order}\;\;\text{model}:C_{t}=C_{o}\;\exp\left(-kt\right)\text{,}$$

(3) $$\text{Weibull}\;\;\text{model}:\frac{C_{t}}{C_{o}}=\exp\left[-{\left(\frac{t}{\alpha }\right)}^{\beta }\right]$$

and

(4) $$\text{Fractional}\;\text{conversion}\;\;\text{model}:\frac{C_{t}-C_{f}}{C_{o}-C_{f}}=\exp\left(-kt\right)\text{.}$$

#### 2.5.3 Functional group determination

The Fourier-transform infrared spectroscopy (FTIR) is an analytical technique used to identify organic material structures and deformation by measuring the intensity of transmitted or reflected light in the organic material sample as a function of its wavelength or wavenumber. It is used to obtain the infrared spectrum of absorption or emission of the chili pepper's bioactive compounds during solar drying [30]. The presence of functional groups (chemical derivatization) such as epoxide, hydroxyl, and carbonyl groups was investigated using FTIR spectroscopy (JASCO FT/IR- 6600, Japan) in a wave number range of 400–4000 cm^−1^. The 1:100 g sample to KBr (Potassium bromide) ratio was used and grounded uniformly using a mortar and pestle for palletization and then absorption spectra were recorded under this wavenumber range.

#### 2.5.4 Capsaicinoid determination

The capsaicinoids were extracted from the freshly harvested and dried chili peppers using the AI Othman et al. method with slight amendments [10]. About 4 g of dried chili pepper was treated with 25 mL of absolute ethanol at 65 °C for 3 h contact time in a water bath with continuous stirring. The final samples were removed from the bath and cooled to room temperature. The supernatant layer of each sample (25 mL) was filtered through a 0.45 μm filter paper and the ethanol leftover was evaporated overnight at room temperature. The extracted sample was stored at 4 °C in a refrigerator until analysis was commenced.

**Standard Stock Solution Preparation**: 10 mg dihydrocapsaicin or capsaicin was dissolved with acetonitrile up to 10 mL to obtain a stock solution of 100 mg/mL. The mixture was then filtered using a 0.45 μm pore size filter paper (Whatman, 7404 - 004) and diluted afterwards as required.

**Fig. 3:**
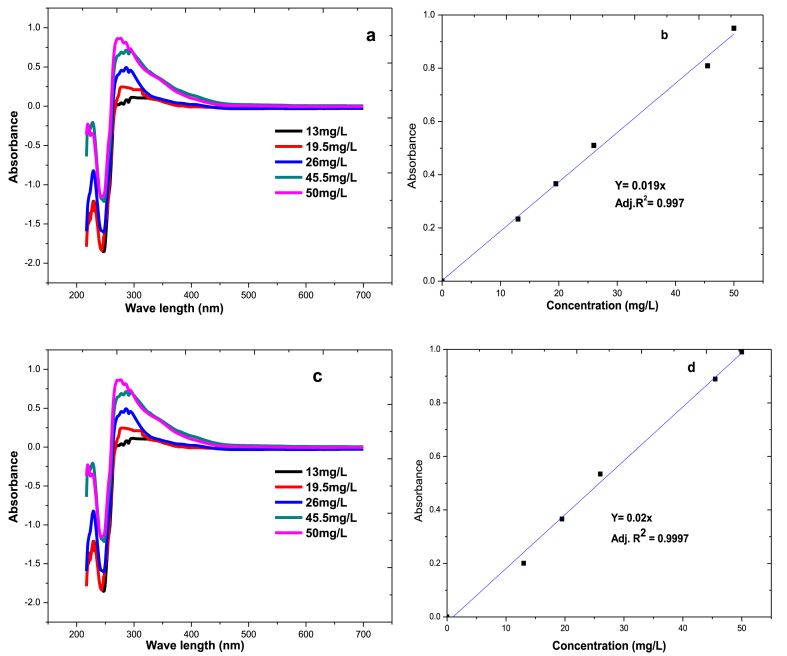
UV–Vis spectra of different concentrations of (a) capsaicin standard, (b) capsaicin standard calibration curve, (c) dihydrocapsaicin standard and (d) dihydrocapsaicin standard calibration curve

**Calibration curve**: linearity tests were carried out for dihydrocapsaicin and capsaicin at five standard solution concentrations (325 μL, 487.5 μL, 650 μL, 1140 μL, and 1250 μL of stock solutions of capsaicin) as shown in Fig. 3. The solution was pipetted to five separate 10 ml volumetric flasks and then filled up to the mark with acetonitrile. From these, 13 ppm, 19.5 ppm, 26 ppm, 45.5 ppm, and 50 ppm of capsaicin concentration was taken and a similar procedure was performed for the dihydrocapsaicin solution. Capsaicinoids are the compounds that are responsible for the spicy flavour, aroma and pungency of the chili peppers. Most of the absorption of capsaicinoids is found between 200 and 350 nm in the UV region of the spectrum since these obey the Beer-Lambert law. The maximum wavelengths of capsaicin and dihydrocapsaicin were found at 278 nm and 225 nm, respectively, as shown in Fig. 3 a & c.

Their corresponding standard calibration curves, to test the linearity, were shown in Fig. 3 b & d. The spectrophotometric calibration curve exhibited a linear response between 13 ***μ***g/mL and 50 ***μ***g/mL and the characteristic equation was *y* = 0.024*x* with a correlation coefficient of 0.997 for capsaicin and *y* = 0.05*x* with a correlation coefficient of 0.999 for dihydrocapsaicin.

**Spectrophotometric Method**: The prepared samples were scanned by a UV–Vis spectrometer (PerkinElmer, Lambda 35, Singapore) to find the appropriate wavelength using a 1 cm quartz cell and λmax = 278 nm was selected as the wavelength of detection. Then, the levels of capsaicinoid content in the chili pepper solutions were determined using the absorbance values of capsaicin standard solutions at 278 nm wavelength. Taking into consideration the degree of purity, dilution factor and molecular mass (capsaicin 305.41 g/mol; dihydrocapsaicin 307.43 g/mol), the molar absorption of each standard capsaicinoid was determined, using the Beer-Lambert law using Eq. (5):

(5) A=ε×b×c.

The limit of detection (LOD) and limit of quantification (LOQ) were determined based on the standard deviation of the y-intercepts of the regression lines from the calibration curve as in Eqs. (6), (7) [31]:

(6) $$LOD_{calibration}=3.3*\left(\frac{\delta }{S}\right)\text{,}$$

(7) $$LOQ_{calibration}=10*\left(\frac{\delta }{S}\right)\text{.}$$

#### 2.5.5 Pungency

Capsaicinoid compounds of chili pepper such as capsaicin (CAP), dihydrocapsaicin (DC), nordihydrocapsaicin (NDC), homocapsaicin (HCAP1-2), and homodihydrocapsaicin (HDC1-2) were converted to a Scoville heat unit (SHU) to get a pungency by multiplying their concentration with their heat values, which are 9.3 for nordihydrocapsaicin, 8.6 for both homocapsaicin and homodihydrocapsaicin, and 16.1 for both capsaicin and dihydrocapsaicin. Afterwards, the Scoville heat unit (SHU) of the chili pepper was evaluated using Eq. (8) [32]:

(8) $$SHU=\left(CAP+DC\right)\times 16.1+NDC\times 9.3+\left(HCAP_{1-2}+HDC_{1-2}\right)\times 8.6\text{.}$$

The pungency was determined considering only the dominant ones, i.e. capsaicin and dihydrocapsaicin.

#### 2.5.6 Ascorbic acid (vitamin C) determination

**Sample extraction:** Sample extraction and vitamin C determination were carried out following the methods proposed by Kapur et al. [33]. Chili pepper samples were extracted by grinding 10 g of sample using a mortar and pestle and then the samples were mixed with 50 mL of a 5 % metaphosphoric and acetic acid solution, put into a 250 mL conical flask and filled with 50 mL of a phosphoric acid solution. Finally, the solutions were filtered using a Whatman filter paper.

**Standard ascorbic acid solution**: The acetic acid amount of 50 mg was mixed with 100 mL of purified water to obtain a standard ascorbic acid stock solution of 500 μg/mL.

**Fig. 4:**
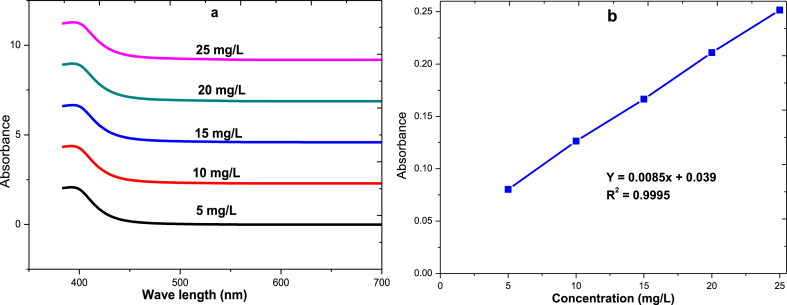
UV–Vis spectra of different concentrations for the determination of vitamin C amount, (a) L-ascorbic acid standard, (b) L-ascorbic acid standard calibration curve

**Standard calibration curve**: Calibration curves at ascorbic acid concentrations of 5, 10, 15, 20, 25 μg/ml were prepared through a proper dilution method as shown in Fig. 4. The absorbance of all standards (converted to coloured complex) was taken to construct a calibration curve as shown in Fig. 4 b.

**Maximum absorption wavelength**: To determine the maximum absorption wavelength, standard solutions of ascorbic acid with a concentration of 500 mg/L were prepared and then the spectrum of the 2, 4-DNPH solution was scanned from 200 nm to 700 nm using a PerkinElmer spectrophotometer. The maximum absorption wavelength was found at 398 nm for all standard concentrations (Fig. 4 a).

**Spectrophotometric method procedure**: Small droplets of a bromine solution were added to the chili pepper sample solution, after which a few droplets of a thiourea solution was added to remove the excess of bromine. One millilitre of a 2, 4 DNPH (2, 4-Dinitrophenylhydrazine) solution was added to the sample solution for all standard calibration curves (5, 10, 15, 20 and 25 μg/mL). Due to the 2, 4 DNPH solution, a coupling reaction occurs and to complete this reaction, all solutions were kept at 37 °C for 3 h after being cooled in an ice bath. Finally, 5 mL of H_2_SO_4_ (85 %) was added which resulted in coloured solutions and the absorbance was measured at a wavelength of 521 nm using a UV–Vis spectrometer (PerkinElmer, Lambda 35, Singapore).

### 2.6 Data analysis

All experimental data were processed using SPSS (a statistical package) and subjected to an analysis of variance (one way and two way ANOVA). A ‘homogeneity of variances’ and a Tukey's Honestly Significant Difference (HSD) test were performed at a 5 % level of significance. The drying model and colour degradation fittings were carried out using Excel to fit the model to the experimental data. Data plots were made using the origin pro 8 data analysis software. The coefﬁcient of determination (R^2^), reduced chi-square (ꭓ^2^), mean biased error (MBE), root mean square error (RMSE) and model constants were determined for all models. The best fit was selected based on the condition of high values of R^2^ and low values of MBE, ꭓ^2,^ and RMSE, where R^2^, ꭓ^2^, MBE, and RMSE were calculated using Eqs. (9), (10), (11), (12) [34].

(9) $$R^{2}=1-\frac{{\sum_{i=1}^{N}\left({\text{MR}}_{\text{pre},i}-\;{\text{MR}}_{\exp,i}\right)}^{2}}{\sum_{i=1}^{N}{\left({\bar{MR}}_{\text{pre},i}-\;{\text{MR}}_{\exp,i}\right)}^{2}}\text{,}$$

(10) $$\chi^{2}=\frac{\Sigma_{i=1}^{N}{\left(MR_{\exp,i}-MR_{\text{pre},i}\right)}^{2}}{N-z}\text{,}$$

(11) $$\text{MBE}=\frac{\Sigma_{i=1}^{N}\left(MR_{\text{pre},i}-MR_{\exp,i}\right)}{N}$$

and

(12) $$\text{RMSE}=\sqrt{\frac{\sum_{i=1}^{N}{\left(M_{\exp,i}-\;M_{\text{pre},i}\right)}^{2}}{N}}\text{.}$$

## 3 Results and discussion

### 3.1 Characterization of solar tunnel dried chili pepper quality parameters

Dried food quality evaluation is the most important parameter during the solar drying process to make the product more competitive in national and international markets since some bioactive compounds might be degraded during drying. All the quality parameters described above were evaluated at a medium chili layer density for both chili varieties.

#### 3.1.1 Surface colour degradation and kinetic modelling

**Fig. 5:**
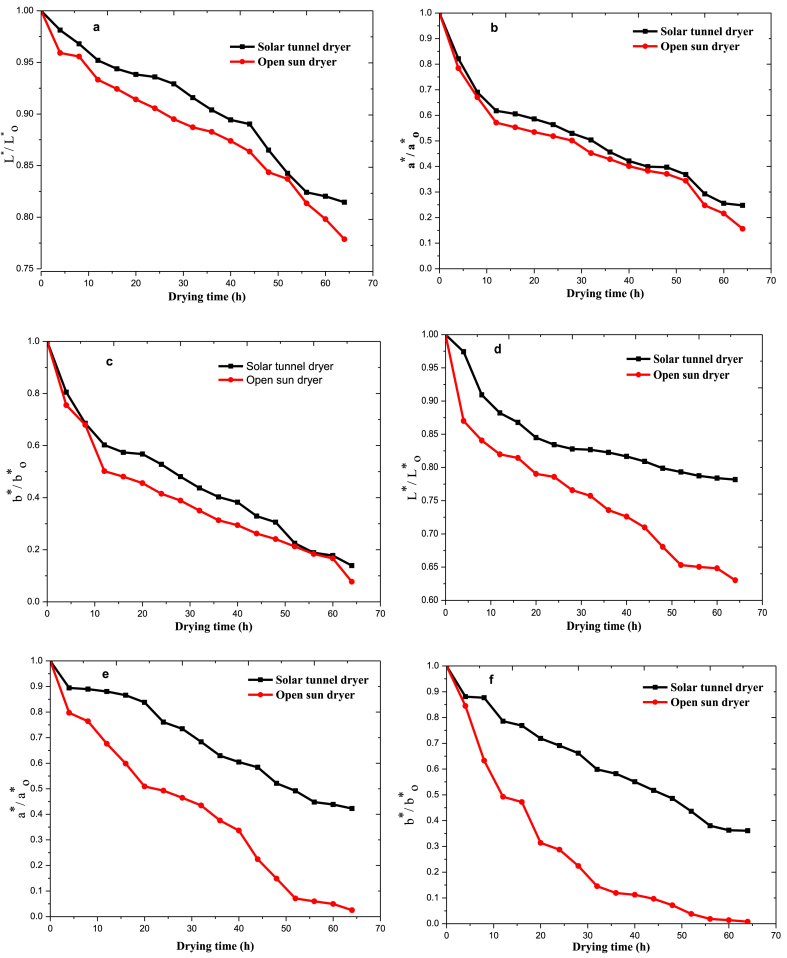
Surface colour change kinetics of chili peppers during solar tunnel and open sun drying, (a–c) Mereko Fana (MF) chili variety and (d–f) Bako Local (BL) chili variety.

The stability and suitability of dried foods are measured through the extent of colour degradation during thermal processing. The freshly harvested chili pepper colour values of L*_o,_ a*_o_, and b*_o_ were 29.33, 12.87, and 8.05, for *Mareko Fana* whereas for *Bako* Local chili variety, it was 35.45, 31.57, and 16.86, respectively. As illustrated in Fig. 5, the L*, a*, and b* colour ratio evolutions clearly demonstrated an exponential decrease during the solar tunnel and open sun drying processes. This is due to the degradation of the carotenoid pigment and the increment of browning compounds through a Maillard reaction in the chili peppers [35]. When compared to the solar tunnel drier, open sun drying was found to have a considerably (p < 0.5) greater rate of colour loss. This suggests that because of its indirect heat source, the solar tunnel chili dryer was somewhat more effective at maintaining the quality characteristics of chili peppers. According to Fig. 5, it was evident that during both solar tunnel and open sun drying for all chili varieties, yellowness experienced the most colour degradation, followed by redness and lightness. This is comparable to the findings of Minguez-Mosquera et al. [36]. The degree of sensitivity for colour degradation was more pronounced for BL than for MF in both drying types. This might be due to the morphological nature of the variants to retain the quality attributes. Similar results were found by Yang et al. [27] and Tamkaew et al. [37]. Ergüneş et al. also reported that chemical pretreatment highly maintained the quality of the chili pepper during greenhouse and open sun drying [38]. The experimental colour data (L*, a*, and b*) were fitted to the zero-order, first-order, Weibull, and fractional conversion models of chili pepper dried in solar tunnel dryer and the associated kinetic parameters are shown in Table 1.

**Table 1:** Values of the drying constants and coeﬃcients of diﬀerent models determined through regression for the chili peppers.

Chili variety	Colour change model	Colour parameter	Coefficients	R^2^	ꭓ^2^	MBE	RMSE
MF	Zero-order model	L*	k = 0.0029	0.9738	8.67E-05	0.0008	0.0090
		a*	k = 0.0137	0.9333	0.0138	0.0513	0.1138
		b*	k = 0.0153	0.9524	0.0127	0.0492	0.1094
	First-order model						
		L*	k = 0.0031	0.9694	0.0001	0.0002	0.0098
		a*	k = 0.0232	0.9767	0.0048	0.0156	0.0671
		b*	k = 0.0281	0.9886	0.0030	0.0104	0.0535
	Weibull model						
		L*	α = 300, β = 1	0.9523	0.0002	0.0062	0.0123
		a*	α = 40, β = 1	0.8651	0.0053	0.0061	0.0708
		b*	α = 50, β = 1	0.9451	0.0143	0.1081	0.1160
	Fractional conversion model						
		L*	k = 0.0031	0.9694	0.0001	0.0002	0.0098
		a*	k = 0.0232	0.9768	0.0048	0.0156	0.0671
		b*	k = 0.0232	0.9768	0.0048	0.0156	0.0671
BL	Zero-order model						
		L*	k = 0.0043	0.5625	0.0019	0.0173	0.0428
		a*	k = 0.0096	0.9819	0.0006	0.0052	0.0243
		b*	k = 0.0111	0.9840	0.0020	0.0185	0.0429
	First-order model						
		L*	k = 0.0049	0.9641	0.0015	0.0137	0.0382
		a*	k = 0.0128	0.9705	0.0010	0.002	0.0311
		b*	k = 0.0159	0.9956	0.0005	0.0041	0.0225
	Weibull model						
		L*	α = 220, β = 1	0.6426	0.0017	0.0234	0.0398
		a*	α = 100, β = 1	0.8675	0.0050	0.0527	0.0686
		b*	α = 50, β = 1	0.9545	0.0055	0.0581	0.0709
	Fractional conversion model						
		L*	k = 0.0159	0.6411	0.0015	0.0137	0.0382
		a*	k = 0.0128	0.9705	0.0010	0.0020	0.0311
		b*	k = 0.0159	0.9956	0.0005	0.0040	0.0225

As shown in Table 1, the first order model gave a higher correlation coefficient (R^2^ > 0.96) and lower standard error (ꭓ^2^ < 0.0048, MBE <0.0156 and RMSS <0.0671) as compared with other colour change kinetic models, which indicated the suitability of the model for predicting the lightness, redness and yellowness kinetics in solar drying for both varieties. The fractional conversion model was the second most suitable model to fit the experimental data for both chili varieties. Yang et al. [27] reported that the Weibull model is the best fit for chili pepper, which indicated that colour variation depends on variety of fruits and vegetables.

#### 3.1.2 Functional groups of chili pepper

**Fig. 6:**
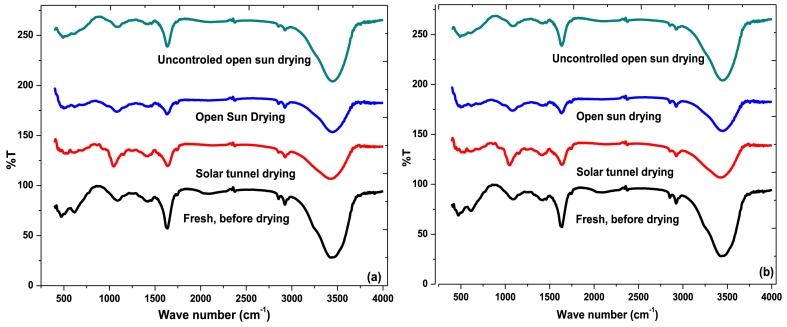
FTIR spectral analysis at different drying methods, (a) *Mereko Fana* chili variety and (b) *Bako Local* chili variety

FTIR spectra of solar tunnel and open sun dried MF and BL chili varieties are presented in Fig. 6a and b, respectively. The spectral scanning range was 400–4000 cm ^−1^ for all varieties. A number of adsorption peaks can be observed, indicating the complex nature of the chili pepper composition. The FTIR spectra also show that the broad peak around 3400–3500 cm^−1^ indicates a functional group of OH stretching vibration modes with a broad and strong intensity, which represents compounds of alcohols and phenols, in general amino acids [30]. The peak around 2850-3000 m^−1^ indicates a C-H stretching vibration with strong intensity, which represents the structure of carboxylic acids [39]. The peak around 2225-2400 cm^−1^ indicates a C ≡ N stretching vibration with medium intensity, which represents nitriles. The peak between 1630 and 1680 m^−1^ indicates the presence of a C-O and OH group stretching vibration functional group with a medium strong intensity, responsible for polyphenols [30]. The peak between 1040 and 1085 cm^−1^ indicates an O-C stretching vibration with medium strong intensity in carboxylic acids or derivatives and esters. The peak around 650-725 cm^−1^ indicates a C-H out of plane bending vibration with a medium intensity in aromatic compounds like alkenes. Finally, the peak around 420-500 cm^−1^ indicates a C-Cl stretching vibration with strong intensity in alkyl-chlorides or alkyl-halides that give sweetness to the chili pepper [40].

Fig. 6 shows the amount of volatile aromatic compounds lost from the chili peppers at wavenumbers between 650 and 725 cm^−1^ in open sun drying. However, solar tunnel drying preserved these compounds for both varieties. These compounds might be responsible for the odour of the chili pepper. Moreover, alkyl-chlorides or alkyl-halides at wavenumbers between 420 and 500 cm^−1^ are removed from both solar tunnel and open sun drying due to their high volatileness. The peaks of carboxylic acids and derivatives and esters became high in the solar tunnel dryer as compared to fresh chili peppers. However, the peak of the nitriles functional group is reduced in all drying modes as compared to fresh chili peppers. The OH and C-O group might represent the functional group of ascorbic acid or vitamin C and capsaicinoids. The peak reduction of OH and C-O groups was more significant (P < 0.05) in open sun drying as compared to solar tunnel drying. This indicated the reduction of vitamin C during the drying process for both chili varieties. In an uncontrolled open sun drying, chili pepper holds more moisture during night, which might enable it to sustain OH and C-O groups. However, open sun drying releases more aromatic compounds, which could be responsible for the quality of the chili peppers.

#### 3.1.3 Capsaicinoid and pungency of chili pepper

Capsaicinoids are responsible for the aroma, pungency and spicy flavour of the chili peppers.

**Molar Absorptivity Coefficient of the Standard Solution**: From the Beer-Lambert law, the molar absorptivity coefficient of pure standard capsaicin was ***ε***_278_ = 8229.5 M^−1^ cm^−1^ and ***ε***_278_ = 8283.9 M^−1^ cm^−1^ for dihydrocapsaicin. The LOD and LOQ were found to be 4.25 and 12.88 ppm for capsaicin and 0.17 and 0.52 ppm for dihydrocapsaicin. The results indicate an excellent recovery ranging from 99.89 % to 100.09 % for capsaicin and 49.03 %–87.35 % for dihydrocapsaicin, which indicates the reliability of the applied method in the spectrophotometer analysis.

**Fig. 7:**
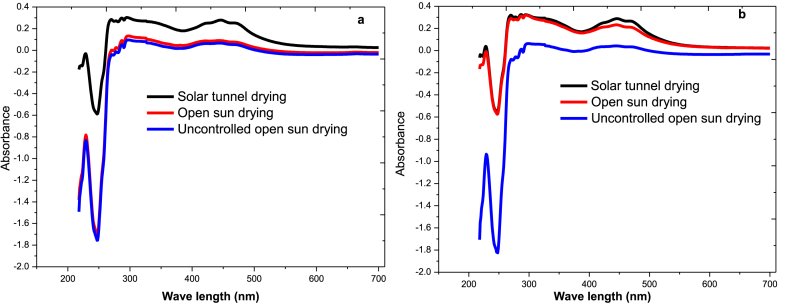
UV–Vis spectra of different drying mechanism of chili varieties of a) *Mareko Fana* chili variety, b) *Bako Local* chili variety

**Chili pepper capsaicinoid**: The extracts of chili pepper dried in different modalities (solar tunnel and open sun drying) were scanned in a spectrophotometer to evaluate their fine structure as shown in Fig. 7. The results indicate that the dried chili pepper has maximum wavelength detections of capsaicin and dihydrocapsaicin at 278 nm and 225 nm, respectively, just like the standard capsaicinoids.

Since standard capsaicinoid is pure white, it does not contain other components such as carotene or vitamins and only shows a fine structure of capsaicinoid as shown in the methodology section 2.4.4 (Fig. 3a). However, dried chili has different components besides capsaicinoid such as carotenes, vitamins, and other bioactive compounds. As shown in Fig. 7a and b, the peaks around 450–465 nm were carotenes, which are responsible for the chili pepper colour. Reports indicated that carotenes are found mostly at wavelengths of 450 nm–460 nm [41].

The concentrations of (dihydro)capsaicin and Scoville heat units (SHU) are presented in Table 2. The results indicate that capsaicin, dihydrocapsaicin and total capsaicinoid levels of the chili pepper dried in different drying modalities have statistically significant differences (P < 0.05) (Table 2) for both varieties. The amount of dihydrocapsaicin and capsaicin from the chili peppers significantly varies (p < 0.05). It ranges from 10,172 μg/kg - 16,313 μg/kg and 16,676 μg/kg - 27,189 μg/kg for both dihydrocapsaicin and capsaicin, respectively. Accumulation of capsaicinoid differs from variety to variety at high drying temperatures as it was observed that capsaicinoid and SHU decreased with increasing drying temperature. Low contents of capsaicinoid and SHU were found in the uncontrolled solar dried chili peppers for both varieties. The percentage of loss of the MF chili variety in solar tunnel, open sun and uncontrolled open sun drying was 3.4 %, 14.8 % and 38.3 %, respectively, while for the BL variety, the values were 1.8 %, 4.4 % and 13.6 %, respectively. The capsaicinoid of MF chili was more vulnerable than the one of BL chili towards drying temperature and drying mode. It was also observed that solar tunnel drying preserves more capsacinoid content compared to open sun drying. González-Zamora et al. [42] reported that high temperatures showed a negative effect on the accumulation of capsaicinoid in certain varieties of chili peppers, such as Jalapeño and De árbol and the loses were 61.5 % and 32.5 % of total capsaicinoid content, respectively due to oxidation of the chili pepper at high temperature. However, they reported that the Serrano pepper variety contained high concentrations of capsaicinoid under high drying temperatures. Topuz and Ozdemir [43] also reported that sun-dried Turkish paprika chili lost 24.6 % of the capsaicin content during drying for 5–7 days, while oven-dried Turkish paprika chili, dehydrated at 70 °C for 90 min, lost 21.5 % of the capsaicin content. Yaldiz et al. [44] also reported that the fruit capsaicin content of different Capsicum species reduced with increasing drying temperatures. On the contrary, there is also an argument comparing the capsaicin content in fresh and dried chili peppers. Capsaicinoid increased in dried chili peppers since dry matter increased during solar and open sun drying processes [45,46].

**Table 2:** Concentrations of capsaicin (dihydro) and Scoville heat units (SHU) of chili peppers.

Chili Variety	Drying mode	Capsaicin (μg/kg)	Dihydrocapsaicin (μg/kg)	Total capsaicinoid (μg/kg)	SHU	Levels of pungency
MF	Fresh, before drying	27189.000	16313.400	43502.400	700388.640	very highly pungent
	Solar tunnel dried	26256.700	15750.000	42006.700	676307.870	very highly pungent
	Open sun dried	23175.600	13905.000	37080.600	596997.660	very highly pungent
	Uncontrolled open sun dried	16675.600	10172.000	26847.600	432246.360	very highly pungent
BL	Fresh, before drying	23284.000	13970.400	37254.400	599795.840	very highly pungent
	Solar tunnel dried	23014.000	13578.000	36592.000	589131.200	very highly pungent
	Open sun dried	22689.000	12933.000	35622.000	573514.200	very highly pungent
	Uncontrolled open sun dried	20622.000	11548.000	32170.000	517937.000	very highly pungent

It is observed that (dihydro)capsaicin was primarily responsible for the SHU rating for both chili varieties (Table 2). Thus, the MF chili variety gave quite a high SHU due to its higher content of capsaicinoid compared to the BL chili variety (Table 2). Uncontrolled solar drying gave the lowest SHU due to a lower content of capsaicinoid compared to controlled open sun drying. The degree of pungency is determined based on the values of capsaicinoid and SHU. It was observed that similar pungency levels were found for all drying modes, although the chili pepper lost some compounds during drying. Both chili pepper cultivars had SHUs between 432246.36 and 700388.64, which indicates that they are extremely hot peppers. (Table 2). Similar SHU results have been reported in other genotype chili peppers [47]. Consequently, depending on their respective levels of capsaicin, the solar-dried BL and MF chili types can both be useful sources of capsaicin for application in various sectors, including the pharmaceutical industry.

#### 3.1.4 Ascorbic acid (vitamin C) of chili pepper

**Fig. 8:**
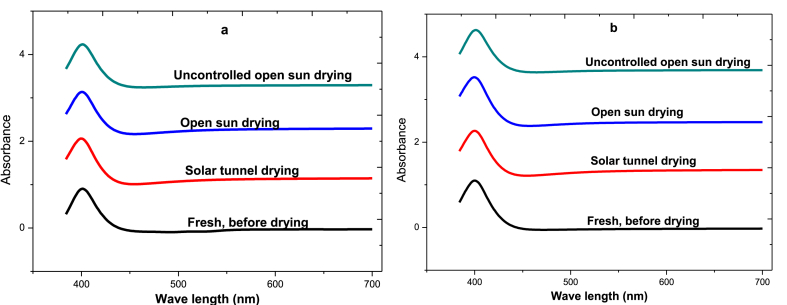
UV–Vis spectral of different drying mechanism of chili varieties to determine vitamin C amounts, a) Mareko Fana chili variety, b) Bako Local chili variety

The ascorbic acid standard calibration curve has been determined using a series of solutions (5–25 mg/L) prepared from a 500 ppm stock solution of L-ascorbic acid. The LOD and LOQ of ascorbic acid were 0.95 μg/mL and 7.03 μg/mL, respectively. The findings showed a good recovery for ascorbic acid, ranging from 98.7 % to 102.98 %, demonstrating the validity of the technique used in the spectrophotometer study. The extracts of chili pepper dried in different modes (solar tunnel and open sun drying) were scanned in a spectrophotometer to evaluate their fine structure as shown in Fig. 8 for both chili pepper varieties. According to the results, the dried chili pepper exhibited the highest ascorbic acid wavelength detection at 398 nm, which was comparable to the standard structure of L-ascorbic acid for both chili varieties.

From the plot of the standard concentration versus corresponding absorbance, the ascorbic acid concentrations of both chili pepper varieties were determined at a wavelength of 398 nm as presented in Table 3. The ascorbic acid content of the freshly harvested chili pepper varied from 10.4 mg/L to 11.1 mg/L for both varieties. By the applied spectrophotometer method, only L-ascorbic acid was found and ascorbic acid concentration significantly (*p* < 0.001) decreased during the drying process. In the solar tunnel, controlled open sun, and uncontrolled open sun drying, the MF variety lost 29, 39, and 90 % of its ascorbic acid, while the BL variety lost 23, 33, and 92 %. It was observed that a minimum loss of ascorbic acids (29–33 %) was recorded in solar tunnel drying related to controlled open sun drying for both varieties.

**Table 3:** Concentrations of ascorbic acid of chili peppers at different drying mechanism.

Chili variety	Drying method	Absorption	Ascorbic acid concentration (ppm)	Ascorbic acid Retention (%)
MF	Fresh, before drying (FBD)	0.127	10.365	100
	Solar tunnel dried(STD)	0.102	7.353	71
	Open sun dried (OSD)	0.100	7.176	69
	Uncontrolled open sun dried (UOSD)	0.030	1.059	10
BL				
	Fresh, before drying (FBD)	0.133	11.059	100
	Solar tunnel dried (STD)	0.112	8.529	77
	Open sun dried (OSD)	0.102	7.424	67
	Uncontrolled open sun dried (UOSD)	0.046	0.871	8

The retention of ascorbic acid in uncontrolled open sun drying was in the range of 8–10 % of the initial value for both varieties. This indicates that the degradation rate of vitamin C was higher in an uncontrolled open sun drying process compared to the solar tunnel and controlled open sun drying. Similar reports have been seen at different drying temperatures and modes [48]. The uncontrolled open sun drying represents conventional drying systems that farmers use widely in the production areas in which chilies are spread on the ground overnight until dried, which results in high bioactive compound losses. Thus, solar tunnel chili drying is more advantageous over open sun drying mechanism with respect to ascorbic acid retention.

#### 3.1.5 Comparison of solar chili dryers

Different parameter comparisons of solar chili dryers were carried out and the comparison results are presented in Table 4. The comparison result showed that the double stage solar chili dryer has a great impact on the preservation of quality attributes of chili pepper during the drying process compared to the other drying process.

**Table 4:** Comparisons of different solar drying mechanism.

Dryer type	Parameters	Country	Reference
Open sun drying	Drying time is more than 91 h, low grade quality	Anywhere	[49]
Solar cabinet	Drying time is 57 h, vitamin C content is 23.99 mg/100g, final moisture content is 4.31–4.88 %	Sumatera, Utara, Indonesia	[50]
Solar tunnel	Drying time is 41–46 h, final moisture content is 14 %	Bogra, Bangladesh	[51]
Solar cabinet	Drying time is 10–30 h for sliced chili, Vitamin C content is 13.75–24.07 mg/100g, final moisture content is 6–9 h	Sumatera, Indonesia	[52]
Forced convection indirect solar cabinet	Final moisture content is 10 % (wb), drying time is 33 h, collector, drying system, and pickup efficiency ere 28 %, 13 %, and 45 %, respectively	Malaysia	[53]
Double-pass solar cabinet drier	Drying time is 32 and 73 h, final moisture content is 10 %, overall drying efficiency is 24.04 %, good ASTA colour value	Prague, Czech Republic	[54]
Solar Hybrid Greenhouse Dryer	Final moisture content is 18.67 % (wb), The organoleptic properties such as texture, colour and flavour of red chili (presence of functional groups by FTIR analysis) were retained by greenhouse drying	India	[55]
Stand-alone solar photovoltaic cabinet dryer	Final moisture level is 0.030 (d b.), drying time is 21 and 37 h, thermal efficiency is 31.37 %, overall thermal efficiency is 64.16 %, payback period is 1.74 year	North-East India	[56]
Solar cabinet with phase change materials	Average drying rate improved h from 0.40 to 0.44 kg/kg-h to 0.40–0.51 kg/kg-h, total phenols (2.43 ± 0.26 mg GAE/g DW, total flavonoids (0.68 ± 0.16 mg CE/g DW), antioxidant activity (83.72 ± 4.30 % RSA, 5.67 ± 0.30 mg TEAC/g DW in FRAP), ascorbic acid (196.71 ± 1.25 mg/100 g) and total carotenoids (1.40 ± 0.37 mg/g)	India	[57]
Controllable lab-scale hot air dryer	Drying takes about 70 h, final moisture content is 10–12 %, optimal drying temperature and relative humidity were 50 °C and 35 %, respectively	Ethiopia	[58]
Indirect double stage solar tunnel dryer	Final moisture content is 11–12 % (w.b), drying time is 50–80 h, moisture diffusivity is 4.47E−09 m2 s−1, collector efficiency is 66.44 %–76.53 %, overall system efficiency is in the range of 24.12–31.3 %, he CO2 emission was calculated as 53.8 kg/kWh	Ethiopia	[12]
Indirect double stage solar tunnel dryer	The amount of dihydrocapsaicin and capsaicin from the chili peppers ranged from 10,172 μg/kg - 16,313.4 μg/kg and 16,675.6 μg/kg - 27,189 μg/kg, respectively. A minimum loss of ascorbic acids was recorded during solar tunnel drying (7.353–8.529 ppm)	Ethiopia	Current study

## 4 Conclusions

This study evaluated the quality attributes of two chili pepper varieties, *Mareko Fana* (MF) and *Bako Local* (BL), during open sun and solar tunnel drying. The first order model provided a good description of the kinetics of thermal degradation of colour. The second best model to fit the experimental data was the fractional conversion model. Colorimetric analysis proved to be a helpful technique for assessing pigment degradation because a high correlation was observed between the evolution of surface color parameters (L*, a*, and b*) and natural pigment concentration during solar drying. Open-air drying of the chili peppers resulted in a loss of their volatile aromatic components, while solar tunnel drying preserved these compounds. For both chili varieties, it was observed that solar tunnel drying resulted in a minimal loss of ascorbic acids (29–33 %) and other quality metrics when compared to open sun drying. Thus, the solar energy based drying technology is more advantageous than the traditional drying technology in terms of preservation of dried food quality and minimization of postharvest losses.

## Data availability statement

Data included in article/supp. material/referenced in article.

## CRediT authorship contribution statement

**Eshetu Getahun:** Writing – original draft, Visualization, Methodology, Investigation, Formal analysis, Conceptualization. **Mulugeta A.Delele:** Validation, Supervision, Conceptualization. **Nigus Gabbiye:** Validation, Supervision, Project administration. **Solomon Workneh:** Visualization, Validation, Supervision. **Maarten Vanierschot:** Writing – review & editing, Visualization, Validation, Supervision, Project administration, Conceptualization.

## Declaration of competing interest

The authors declare that they have no known competing financial interests or personal relationships that could have appeared to influence the work reported in this paper.
